# Diagnosing the current state of out-of-field teaching in high school science and mathematics

**DOI:** 10.1371/journal.pone.0223186

**Published:** 2019-09-25

**Authors:** Lisa Shah, Cooper Jannuzzo, Taufiq Hassan, Bogdan Gadidov, Herman E. Ray, Gregory T. Rushton

**Affiliations:** 1 Department of Chemistry, Stony Brook University, Stony Brook, New York, United States of America; 2 Department of Statistics and Analytical Science, Kennesaw State University, Kennesaw, Georgia, United States of America; 3 Analytics and Data Science Institute, Kennesaw State University, Kennesaw, Georgia, United States of America; 4 Tennessee STEM Education Center, Middle Tennessee State University, Murfreesboro, TN, United States of America; Universidad Nacional de Educacion a Distancia (UNED), SPAIN

## Abstract

The U.S. government has acknowledged the critical role that teachers play in the production of Science, Technology, Engineering, and Mathematics (STEM) professionals who will drive our nation’s economy. The No Child Left Behind Act of 2001 (NCLB) was passed to improve the quality of education nationwide, in part, by decreasing the number of out-of-field (OOF) teachers. However, the impact of NCLB and related efforts on the current state of OOF teaching in high school science and mathematics has yet to be examined. Our analysis of data from the National Teacher and Principal Survey (NTPS) indicates that from 2003–2016, the proportion of OOF teachers in chemistry and physics has increased, and there has been an increase in the number of students assigned to OOF teachers across subjects. We discuss the societal impact of our results and the critical role that policymakers, school administrators, and academic institutions, particularly university faculty, can play in its solution.

## Introduction

A well-educated STEM workforce is a fundamental pillar of our economy. STEM professions stand at the forefront of innovation and entrepreneurship, outpacing the growth of other jobs in the U.S. by 300 percent [1]. It is essential that highly-proficient STEM majors pursue these demanding careers. With a greater demand for highly-capable STEM students comes a reciprocal need for effective STEM educators.

Content knowledge has been recognized as a significant component of effective teaching and is often indirectly measured by whether a teacher has a major/minor and/or a certification in their subject [2,3]. A major/minor in a particular discipline is widely recognized as a proxy for having had significant coursework offered by STEM departments in the subject area [4–6] and a number of studies have reported that subject-specific coursework has been linked to student learning [7] and more effective instruction [8]. In almost all U.S. states, teaching certifications are awarded to teaching candidates in specific disciplines following satisfactory performance on state licensure exams, which serve to evaluate prospective teachers’ subject-matter knowledge [9,10] and studies have shown that teachers with subject certification positively impact student learning in STEM [11].

Despite these reports, out-of-field (OOF) teaching, or the assignment of a teacher to a subject for which (s)he is not sufficiently qualified, has been documented as a prevalent issue in K12 science and mathematics teaching in the late 1990s and early 2000s. Several reports have cited the negative impact of OOF teachers on student achievement [7,12], and a number of high-profile studies have reported that OOF teachers comprised as much as one-third to one-half of science teachers in the U.S. during this time [4,5,13,14]. Informed by these and related studies, the U.S. federal government issued the No Child Left Behind Act of 2001 (NCLB), a reauthorization of the Elementary and Secondary Education Act of 1965 (ESEA), in an attempt to put an end to out-of-field teaching [15]. One objective of this legislation was to improve the state of out-of-field teaching in the United States, requiring that all teachers be “highly-qualified” to teach by the end of the 2005–2006 academic year by (i) having coursework in their subject area and (ii) passing a teaching certification exam. As a result, national and state agencies have since invested in efforts to recruit and retain highly-qualified educators in K12 classrooms [16–18]. It should be noted that the original licensure requirements under NCLB were loosened by 2006 such that science teachers, in particular, could now be certified in a specific discipline or under broad-field, general science certifications. Further, while these standards were presented as requirements, the consequences or a lack of compliance were not made clear.

What has yet to be established is whether the overall condition of out-of-field teaching in high school science and mathematics in the U.S. has improved since the passage of NCLB almost two decades later. It would be reasonable to hypothesize that positive changes may now be detectable, which would validate legislation and related policies, and inform future decisions in this realm. Additionally, existing studies have not acknowledged the unique nuances of OOF teaching in the sciences. While ‘science’ is often considered a homogenous subject area with respect to staffing, many science teachers and disciplinary experts would likely agree that ‘science’ in more accurately comprised of several distinct disciplines (e.g., biology, chemistry, physics). Apart from differences in foci between disciplines (e.g., living organisms in biology, matter in chemistry), knowledge construction is also reportedly unique to each subject [19,20], such that training in one science discipline is unlikely to qualify one to teach across others [21, 22]. An evaluation of out-of-field teaching across science subjects is needed to inform discipline-specific efforts.

Our study uses data from the National Teacher and Principal Survey (NTPS), the largest, most comprehensive system of surveys regarding K12 districts, schools, and teachers in the U.S. [23], to address this gap in the STEM education literature. The following research question guides our analysis: How has the state of out-of-field teaching across high school science disciplines and mathematics changed with respect to both educators and students in the U.S. since NCLB?

## Materials and methods

### National Teacher and Principal Survey (NTPS)

Administered by the National Center for Education Statistics (NCES), NTPS (formerly Schools and Staffing Survey before 2015) is the largest, most comprehensive system of surveys regarding K12 districts, schools, and teachers in the United States. Data for this work comes from the Teacher Questionnaires administered in 2003–2004, 2007–2008, 2011–2012, and 2015–2016. For each survey year, our analytical focus was on teachers who reported teaching at least one class period at the high school level (i.e., grades 9–12) in chemistry, physics, biology, or mathematics. NCES collects survey data containing individually-identifiable information from survey respondents that are confidential and protected by law. Access to this database is provided through a restricted-access license from NCES, which allows for the use of these data for research and publication. The authors have complied with the terms of service for these data. Using a complex sampling design, these surveys are administered to individual teachers and administrators (e.g., principals) within representative, demographic strata to collect detailed information about topics such as school climate, teacher credentials, and student characteristics. Responses from individuals within this sample are then weighted to account for selection likelihood, reduce bias, and maximize the precision of estimates regarding the larger population. Weights are provided in the survey data and we report only weighted statistics here.

### Classifying respondents as teachers of specific subjects

The survey design allows for each respondent to indicate the subject (s)he teaches for each of up to ten class periods per day. In our analysis, respondents were classified as teachers of a particular subject if (s)he indicated teaching at least one class period in that subject area using the survey codes listed below (Table 1). For example, mathematics teachers indicated teaching at least one class period with one of the codes listed under Mathematics. Using this approach, it is certainly possible for respondents to be classified as teachers of two or more distinct subjects.

**Table 1:** NTPS survey codes used for the assignment of respondents as teachers of specific discipline.

Subject Area	Survey Codes
*Mathematics*	191 Algebra I; 192 Algebra II; 193 Algebra III; 194 Basic and general mathematics; 195 Business and applied math; 196 Calculus and pre-calculus; 198 Geometry; 199 Pre-algebra; 200 Statistics and probability; 201 Trigonometry
*Biology*	211 Biology or life sciences
*Chemistry*	212 Chemistry
*Physics*	217 Physics

### In-field/Out-of-field teachers based on major/minor and certification

Respondents were classified as having an in-field major/minor if they reported having any of the following in the same subject area in which they taught at least one course: major, second major, minor, Master's degree, or PhD. Respondents were classified as having an in-field certification if they reported having any kind of certification valid for grades 9–12 in the same subject area in which they taught at least one course. The survey codes used for both types of qualifications are listed in Table 2 for each subject area.

**Table 2:** NTPS survey codes used for the classification of subject-specific majors/minors and certifications to teacher respondents.

Subject Area	Survey Codes
*Mathematics*	190 Mathematics
*Biology*	211 Biology or life sciences
*Chemistry*	212 Chemistry
*Physics*	217 Physics

Teachers were then classified as in-field or out-of-field based on whether their reported major/minor/degree and/or certification matched the subject area in which they reported teaching as follows (Table 3).

**Table 3:** Working definitions of in-field/out-of-field teachers used in this study.

Qualification Level	Qualifications Held
*In-field*	In-field major/minor AND In-field certification
*Out-of-field (OOF)*	In-field major/minor ONLY
	In-field certification ONLY
	No in-field major/minor AND No in-field certification

### Weighted numbers of teachers and students

Final teacher weights provided by NCES in the survey data are applied to unweighted descriptive teacher counts to yield the reported teacher numbers. For each class period reported, teachers also indicate the number of students enrolled in the class. Using these values, final teacher weights are similarly applied to the unweighted number of students for each individual respondent to generate the reported number of students assigned to a specific group of teachers (e.g., the number of students assigned to in-field chemistry teachers).

### Statistical comparisons of survey estimates

The balanced repeated replication (BRR) approach is used to produce replicate survey weights (which are also included in the dataset) that can be used to more accurately estimate standard errors for each survey response. The coefficient of variation (CV), or the ratio of the standard error (SE) of an estimate to the mean of the estimate, is calculated for each disciplinary sub-group of teachers and students as previously documented by NCES as shown in Eq 1 [24]

$$CV=\frac{\sqrt{(\frac{1}{200}\sum_{r}{\left(Y_{r}-Y\right)}^{2})}}{sample\;estimate}$$ (1)

where Yr is the estimate of Y using the rth set of replicate weights and Y is the mean of the 200 replicates.

Linear trend tests were used to examine longitudinal trends in reported teacher credentials and student assignments to these teachers over time. This was achieved by performing a simple linear regression of annual data points for a given discipline and sub-group (e.g., biology teachers with *only* a certification in biology in each survey year) and using the ratio of the regression coefficient (*β*) and its corresponding standard error (*se*) as the test statistic (*t*). The computed *t* value was then compared with published values of *t* for two-tailed hypothesis testing using a 5% probability of Type I error (i.e., a significance level of .05). In no case were longitudinal trends in teacher or student sub-groups within disciplines statistically significant. This is not unexpected given that there is no reason that changes in estimates from year to year would necessarily follow a linear progression. These data are not explicitly reported and our analyses.

Independent samples *t* tests were performed to examine changes in teacher or student means since NCLB by comparing estimates in the first (i.e., 2003–04) and last (i.e., 2015–16) survey years across subgroups and disciplines. For example, the proportion of chemistry teachers who reported BOTH a major/minor/degree AND certification in chemistry in 2003 was compared to that in 2015 to determine whether these proportions of teachers were statistically different. *t-*statistics were calculated as shown in Eq 2

$$t=\frac{E_{1}-E_{2}}{\sqrt{{se}_{1}^{2}-{se}_{2}^{2}}}$$ (2)

where E_1_ and E_2_ are the two estimates being compared and se_1_ and se_2_ are their corresponding standard errors. The *t* value was then compared with published values of *t* for two-tailed hypothesis testing at a significance level of .05. *t* values ≥ 1.96 (i.e., the critical value of the *t* distribution for a 5 percent level of significance with a large sample size) signify that the estimates being compared are statistical different.

To quantify statistically significant comparisons between the proportions of two different groups of teachers or students (i.e., P_1_ and P_2_) in various survey years, Cohen’s *h* was calculated as a measure of effect size as shown in Eqs 3 and 4 [25].

$$h=\varphi_{1}-\varphi_{2}$$ (3)

$$\varphi_{i}=2*arcsin\sqrt{P_{i}}$$ (4)

## Results

Consistent with metrics used in the OOF teaching literature, we adopt the following working definition of OOF teaching: the assignment of a teacher to a subject for which (s)he does not hold BOTH a university major/minor and a certification in the discipline. Additionally, we categorize in-field teachers as those with both an in-field major/minor and in-field certification. Details regarding survey codes assigned to each category of teachers and disciplines are listed in Table 3.

### Chemistry

**Fig 1:**
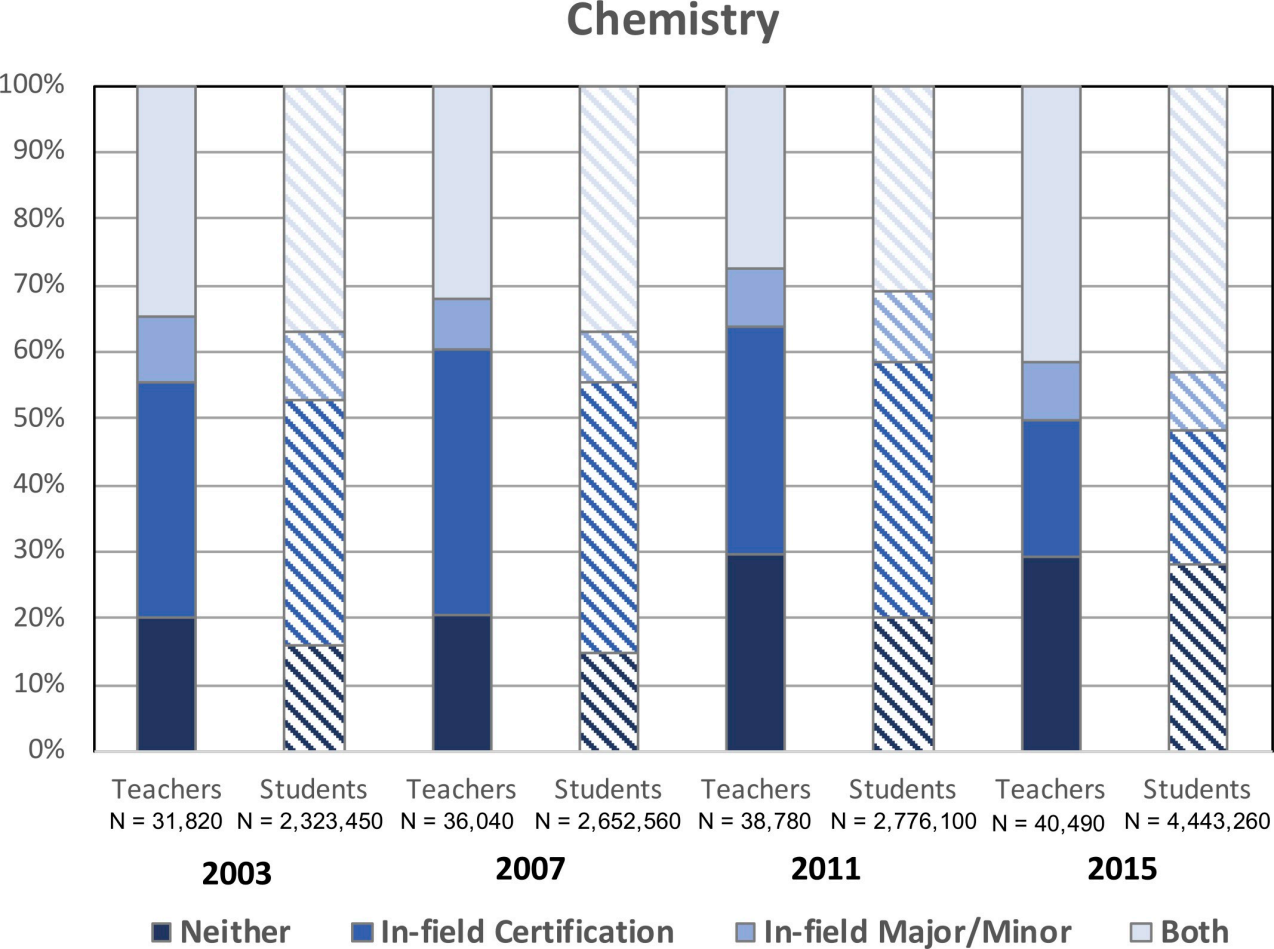
Percentages of in-field and out-of-field high school chemistry teachers and high school chemistry students assigned to these educators from 2003–2015. CV_teachers_ < .013; CV_students_ < .008. SE_teachers_ ≤ 8%; SE_students_ ≤ 5%.

Fig 1 displays the percentage of in-field and OOF high school chemistry teachers and the corresponding percentage of chemistry students assigned to each group of teachers. The percentage of in-field chemistry teachers has increased from approximately 35% in 2003 to 41% in 2015, though the difference is not statistically significant (p > .05, t = 1.4). Among OOF teachers, the percentage of teachers lacking either qualification to teach chemistry (i.e., ‘*Neither*’) has risen significantly from 20% to 29% during this same timeframe (p < .05, t = 2.0, *h* = .19). These increases have been matched by a commensurate decrease in the percentage of chemistry teachers with only in-field certifications (i.e., 35% to 21%) during this timeframe (p < .05, t = 3.5, *h* = -0.31). Additionally, chemistry students have been increasingly assigned to OOF teachers during this timeframe. For example, while 20% of chemistry teachers were teaching without a subject-specific degree/major/minor or certification and were responsible for 16% of chemistry students in 2003, by 2015 these percentages had increased to 29% and 28%, respectively (for students assigned to ‘*Neither*’ chemistry teachers in 2003 and 2015 p < .05, t = 4.8, *h* = 0.29).

### Physics

**Fig 2:**
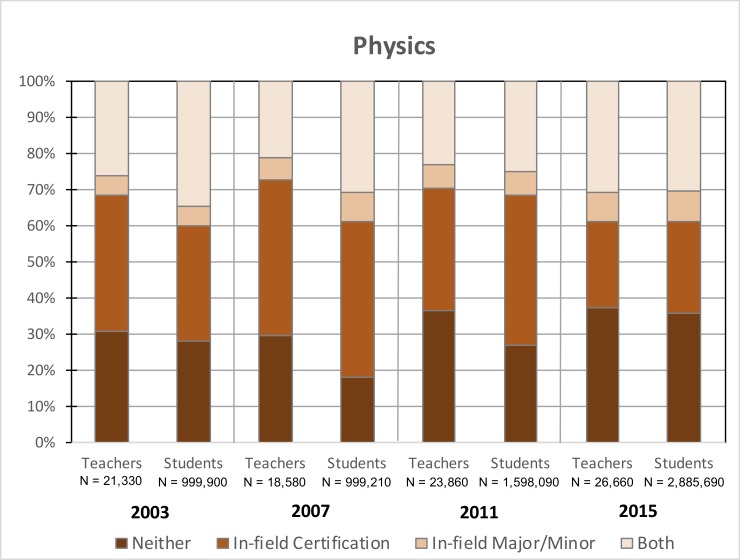
Percentages of in-field and out-of-field high school physics teachers and high school physics students assigned to these educators from 2003–2015. CV teachers < .017; CV students < .011; SE_teachers_ ≤ 10%; SE_students_ ≤ 6%.

Fig 2 depicts the percentage of in-field and out-of-field high school physics teachers as well as the percentage of physics students assigned to each group of teachers. On average, 25% of physics teachers were teaching in-field and 74% were teaching OOF during this time frame. From 2003 to 2015, there was about a 5% increase in in-field physics teachers (i.e., 26% to 31%; p > .05, t = 0.8) though the difference was not statistically significant. Similarly, there was a non-statistically-significant 7% increase in the percentage of teachers with neither qualification (i.e., 31% to 38%; p > .05, t = 0.9). In 2003, in-field physics teachers (26%) reportedly taught a greater fraction of the student population (i.e., 36% of the student population) than did the 31% of physics teachers who reported no teaching qualification in physics (i.e., 30% of the student population). But by 2015, the 31% of in-field teachers were now teaching 30% of the physics student body and the 38% of OOF teachers lacking both types of qualifications were responsible for 36% of physics students (for students assigned to ‘*Both*’ physics teachers in 2003 and 2015, p < .05, t = 2.9, h = -.13; for students assigned to ‘*Neither*’ physics teachers in 2003 and 2015, p < .05, t = 3.8, h = .17). Thus, while in-field physics teachers were responsible for a relatively larger proportion of the physics student body in 2003, by 2015, physics teachers with varying levels of qualifications have been assigned to roughly equivalent numbers of physics students.

### Biology

**Fig 3:**
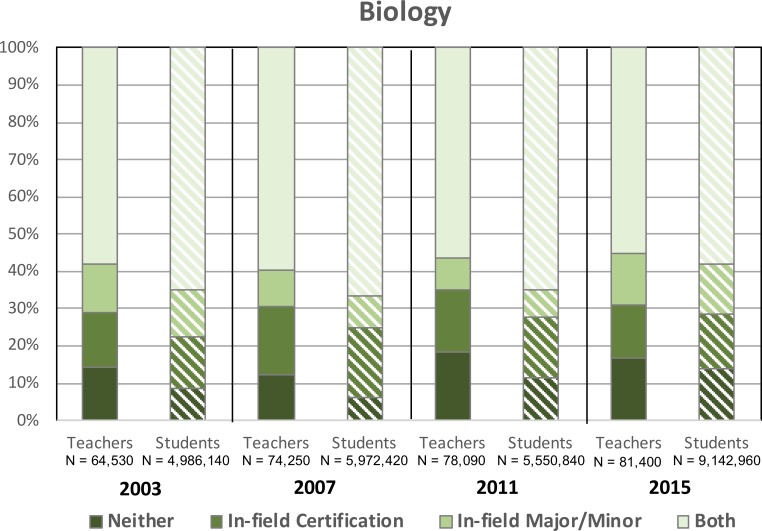
Percentages of in-field and out-of-field high school biology teachers and high school biology students assigned to these educators from 2003–2015. CV teachers < .008; CV students < .002; SE_teachers_ ≤ 5%; SE_students_ ≤ 2%.

Fig 3 displays the proportion of high school biology teachers with varying degree/certification credentials as well as the proportion of biology students assigned to each group of teachers. The relative proportions of biology teachers with varying credentials in each survey year has remained fairly consistent (i.e., linear trend test was not significant AND means for each sub-group in 2003 were not statistically different from that in 2015), with in-field and OOF biology teachers steadily representing approximately 57% and 43% of the workforce, respectively on average (for ‘*Both*’ teachers in 2003 and 2015, p > .05, t = 0.09; for ‘*Neither*’ teachers in 2003 and 2015 p > .05, t = 0.15). A time-frame average of 11% and 16% of biology teachers held just in-field degrees or in-field certifications, respectively (for ‘*In-field degree*’ teachers in 2003 and 2015, p > .05, t = 0.16; for ‘*In-field certification*’ teachers in 2003 and 2015 p > .05, t = 0.08). But while the percentage of biology teachers across qualification levels has not changed significantly, the percentage of students assigned to underqualified educators has increased from 2003 to 2015. For example, while the 14% of ‘*Neither*’ biology teachers were responsible for just 8% of biology students in 2003, by 2015 these percentages were 17% and 14%, respectively (for ‘*Neither*’ biology students in 2003 and 2015, p < .05, t = 4.9, h = .27).

### Mathematics

**Fig 4:**
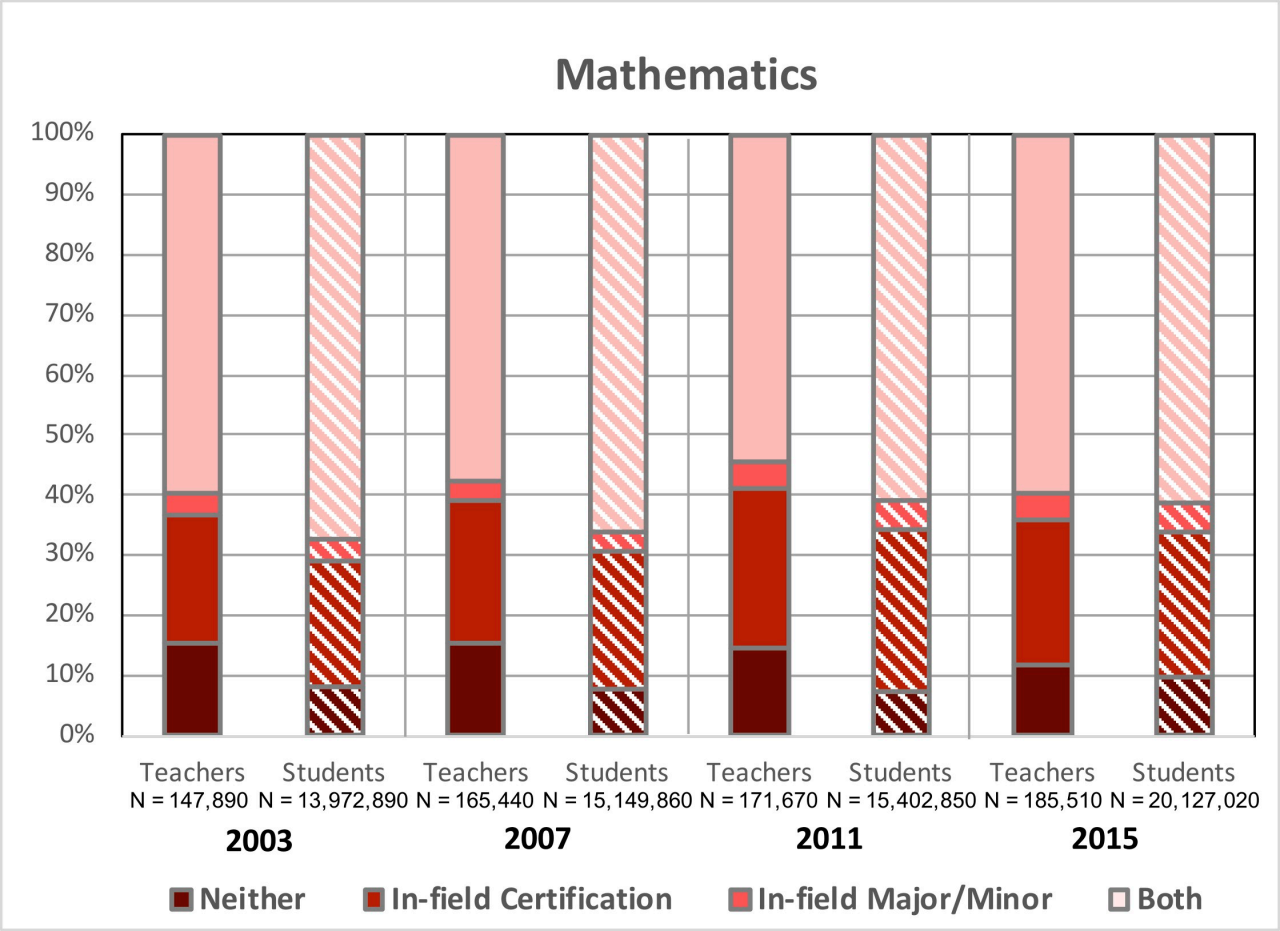
Percentages of in-field and out-of-field high school mathematics teachers and high school mathematics students assigned to these educators from 2003–2015. CV_teachers_ < .008; CV_students_ < .001; SE_teachers_ ≤ 4%; SE_students_ ≤ 1%.

Fig 4 depicts the percentage of high school mathematics teachers with various combinations of in-field degrees/majors/minors and certifications and the percentage of mathematics students assigned to each subset of these teachers. Analogous to the observed trends in the biology teacher population, the relative proportions of mathematics teachers with varying qualifications in each survey year has remained fairly consistent (i.e., linear trend test was not significant AND means for each sub-group in 2003 were not statistically different from that in 2015), In-field mathematics teachers represented approximately 58% of all mathematics teachers during this period (for 2003 and 2015, p > .05, t = 0.11). A time-frame average of 4% and 24% of OOF mathematics teachers held just in-field degrees or in-field certifications, respectively (for ‘*In-field degree*’ teachers in 2003 and 2015, p > .05, t = 0.38; for ‘*In-field certification*’ teachers in 2003 and 2015 p > .05, t = 0.42). An average of 14% of OOF mathematics teachers were teaching without either qualification (in 2003 and 2015, p > .05, t = 0.13). Though the proportions of mathematics teachers across qualification levels has not changed appreciably since 2003, the proportion of mathematics students assigned to teachers with neither qualification has increased. Notably, the roughly 60% of in-field mathematics educators were responsible for 67% of students in 2003, but just 61% of students by 2015 (for ‘*Both*’ mathematics students in 2003 and 2015, p < .05, t = 3.8, h = .23).

## Discussion

Our analysis of NTPS data from 2003–04 to 2015–16 academic years reveals several important findings about the qualifications of our nation’s public high school STEM teachers since the passage of NCLB. Though the effect sizes are small, the proportion of OOF teachers has statistically-significantly increased from 2003 to 2015 among the physics and chemistry workforces. Additionally, the proportion of teachers across qualification levels has remained statistically unchanged among the biology and mathematics workforces, and students across subjects have been increasingly assigned to less qualified teachers. It seems that the goals of NCLB have yet to be realized almost twenty years later and, to a large extent, the situation in our schools has worsened since the early 2000s.

While our data do not allow us to incontrovertibly provide a rationale for our findings, we contend that these results have a number of implications for STEM departments, policymakers, school administrators based on the likely contributors to OOF teaching noted in the literature. First, the recruitment and retention of highly-qualified STEM students into teaching careers has long been documented as a serious issue in K12 STEM education [26, 27] and presumably plays a role in our observed increase in out-of-field teaching (particularly in chemistry and physics). More and more, top students are moving away from careers in STEM teaching toward more lucrative options in professional sectors like healthcare, business, and industry [28]. Given that out-of-field science and mathematics teachers currently comprise 40–60% of educators in each discipline (Figs 1–4), improving approaches to current recruitment and retention strategies is an area in need of attention. Further, a growing percentage of educators across the disciplines analyzed here still lack a major/minor and certification in the subjects they are assigned to teach (Figs 1–4) despite the requirements set forth in NCLB. One important consideration is that the term “highly-qualified” was vaguely defined in the NCLB legislation, requiring that teachers hold a bachelor’s degree and state certification, and demonstrate content knowledge in the subjects they teacher. With actionable power in the hands of individual states [29], this broad definition allowed most teachers to continue to teach subjects in which they were underqualified or even OOF to do so, the results of which are apparent in our data. Additionally, as the 2005–2006 deadline cited in the original legislation approached, the situation was further aggravated, as these standards for highly-qualified, in-field teachers were loosened. Science teachers, in particular, could now be certified in a specific discipline or under broad-field, general science certifications. However, as previously noted, research in this area does not support such decisions. With the 2015 revision to NCLB (i.e., Every Student Succeeds Act, ESSA) increasing the power and flexibility of the states [30], how individual states and local school administrators respond will likely determine whether the condition of OOF teaching in our nation will improve. Efforts to better align related legislation with empirical evidence in existing education literature may be crucial in this regard. For example, the preparation of STEM education scholars (who have the expertise required to make informed decisions regarding K12 policy) for careers in government might be an effective approach to enacting such change. Finally, efforts to support those teachers, whether in-field or out-of-field, who are already in our nation’s classrooms is crucial. Discipline-specific professional development and mentoring may help to strengthen the subject-matter competence and pedagogical content knowledge needed to improve their teaching and, subsequently, student learning [31].
